# Anaesthesia challenges of a parturient with paramyotonia congenita and terminal filum lipoma presenting for labour and caesarean section under epidural anaesthesia – a case report

**DOI:** 10.1186/s12871-021-01262-4

**Published:** 2021-02-18

**Authors:** Wei Shyan Siow, Wan-Ling Alyssa Chiew

**Affiliations:** 1 Changi General Hospital, Singhealth. 2 Simei Street 3, 529889 Singapore, Singapore; 2 National Healthcare Group (NHG) Anaesthesiology Residency, 11 Jalan Tan Tock Seng, 308433 Singapore, Singapore

**Keywords:** Paramyotonia congenita, Terminal Filum Lipoma, Caesarean, Epidural, Case Report

## Abstract

**Background:**

Paramyotonia congenita is a rare autosomal dominant myopathy which presents with periodic weakness due to cold and exercise. It is caused by mutations of the SCN4 gene which encodes the sodium channel in skeletal muscles.

**Case presentation:**

We report a full term obstetric patient with both paramyotonia congenita and terminal filum lipoma who presents for induction of labour followed by an emergency caesarean section performed under epidural anesthesia. Her recovery is subsequently complicated by a 3-day history of postpartum paraparesis attributed to hypokalemic periodic paralysis.

**Conclusion:**

We describe the perioperative anesthesia considerations and challenges in this case with a review of the current literature. This case report highlights the importance of early proactive and collaborative multidisciplinary approach, maintaining normal temperature and electrolytes with a heightened vigilance for muscle-related perioperative complications.

## Background

Paramyotonia congenita (PMC) is classified as a form of periodic paralysis characterized by irregular episodes of flaccid paresis and stiffness exacerbated during exercise or cold. It is a rare autosomal dominant genetic condition with an estimated prevalence of 1:200,000. This condition is due to mutations in the SCN4A gene responsible for encoding skeletal muscle sodium channels, therefore causing dysregulation of sodium ion flows into muscle cells resulting in abnormal contraction and relaxation. [1, 2]

Filum terminale lipomas represent the most common intraspinal lipomas, which are usually incidental findings, and most patients are asymptomatic. However rarely this may result in tethered spinal cord syndrome caused by abnormal tissue attachments and traction of the cord within the spinal column. [3] Symptoms may vary including leg or perineal pains, weakness, sensory deficits, sphincter dysfunction or cutaneous stigmata of spinal dysraphism. Onset of this condition may be precipitated during pregnancy by fetal head compression, positioning during childbirth or the epidural itself.

Currently, there are no case report describing a parturient with PMC and filum terminale lipoma undergoing induction of labour and emergency caesarean section (CS). We describe the perioperative management of a parturient who suffered from prolonged limb weakness, most likely due to hypokalemic periodic paralysis after emergency CS under epidural anaesthesia.

## Case presentation

A 33-year-old primigravida of Malay origin presents for elective induction of labour at full term. In view of the chronic history of symptoms of neckache, backache, lower limb pain and weakness, this patient was extensively worked up and found to have PMC and hypokalemia periodic paralysis. She was also found to have a coexisting history of cervical myelopathy and an incidental spinal cord lipoma extending from filum terminale at L2 to sacrum shown on her magnetic resonance imaging (MRI) spine done in 2010. Otherwise, her other previous investigations were normal including autoimmune serology, cervical muscle electromyography, nerve conduction and transcranial magnetic stimulation studies.

The anesthesia team was involved early on admission for her perioperative care in order to plan for options of labour analgesia and anesthesia should she require a CS. She had frequently experienced cold and exercise-induced muscle cramps and weakness since young and was diagnosed of PMC by her neurologist. She was asymptomatic at the time of assessment with a normal neurological examination. She denied any personal or familial history of anesthesia related complications. Her baseline ECG and routine blood results were unremarkable. On consult with the neurologist, the risks of anesthesia induced rhabdomyolysis and malignant hyperthermia could not be fully excluded should she undergo general anesthesia.

As the MRI findings of the lipoma was dated back in 2010, we advised for a repeat MRI intrapartum to assess the extent of lipoma, however she was not keen. In view of terminal filum lipoma, the neurosurgery team advised that a standard epidural would be relatively safer than a combined spinal epidural or a single shot spinal in order to spare the dura.

The risks and benefits of each labour analgesia options including Entonox, intramuscular pethidine injections, Remifentanil patient-controlled analgesia (PCA) and regional analgesia (combined spinal epidural (CSE) and standard epidural) were discussed. We advised for an early standard epidural but she opted for Entonox initially.

Should she require a Caesarean section, we also preemptively counselled her for regional versus general anesthesia. We highlighted a range of muscle related risks such as hyperkalaemia, muscle weakness, myotonia, rhabdomyolysis and malignant hyperthermia should she undergo a general anesthetic. We also emphasized high risk of nerve injury should she undergo a central neuraxial block.

Eventually, she opted for a standard epidural during her early second stage of labour. An epidural catheter was inserted after 3 attempts in the intervertebral space between L3 and L4 using the loss-of-resistance technique to saline in the sitting position. A test dose of 1 % lidocaine 3mls followed by a maintenance infusion dose of 0.125 % ropivacaine with 2mcg/ml fentanyl at 10ml/hour was instituted according to our hospital protocol. Hypoesthesia was confirmed at T10 dermatome and the epidural was continued uneventfully.

Due to prolonged second stage of labour and failure to progress, an emergency CS was activated. Prior to surgical incision, a satisfactory sensory block level to T4 dermatome to pin-prick test was achieved with an epidural top up of 20ml of 2 % lidocaine with 2ml of 8.4 % sodium bicarbonate and 1:200,000 adrenaline. Her Bromage score was 3. Utmost care was taken to maintain normothermia and prevent shivering. Efforts included ensuring ambient room temperature in theatre, the use of forced air warming blanket, and the use of prewarmed intravenous and cleaning fluids. Perioperative electrolytes were closely monitored to be within normal range. We also ensure that there was a malignant hyperthermia (MH) rescue kit and ventilator is readily available in event she needs to convert to general anaesthesia. A neonatal team was also on standby. An uneventful caesarean section was performed with the delivery of a healthy male baby. In the recovery unit, normothermia was maintained throughout. The patient was monitored for one hour in the operating theatre recovery unit. On discharge to high dependency unit, her bromage score was 2. She was subsequently transferred to a general ward thereafter.

However, on post-operative day 1 she continued to have prolonged bilateral lower limb weakness of bromage score 2 or medical research council grade 1 to 2 out of 5. She had a full return of sensation and her anal tone was intact and denied any saddle anesthesia, urine or faecal incontinence. There was no sign of return of power even till post-operative day 3. The neurologist suggested an urgent post-operative MRI lumbar spine which ruled out any epidural collection or significant spinal canal narrowing, only reporting superficial oedema and contusions in the lower back which was likely related to the recent epidural. It was unlikely to cause the prolonged paralysis.

The neurologist eventually attributed the weakness to hypokalemic periodic paralysis as a diagnosis of exclusion. The weakness coincided with a borderline low potassium level of 3.5–3.7 mmol/L on post operative day 1–3. Throughout we closely monitored her neurology, temperature, glucose, oxygenation and perfusion at all times. Her power returned fully on post-operative day 4 when the potassium levels increased to 4mmol/L with the help of potassium supplementation. She was discharged a day after. With early counselling and continued support from the multiple disciplines throughout, she felt assured and satisfied with her perioperative care in hospital.

## Discussion and conclusions

This case report highlights the importance of an early multidisciplinary approach, need to maintain normal balanced temperature and electrolytes with a heightened vigilance for muscle-related perioperative complications in a parturient with PMC who underwent CS after failed labour and delivery. Our literature search of obstetric PMC reveals case reports by Grace et al. [4] and more recently Najid et al. [5] who described an emergency CS of a parturient with PMC that was done under epidural anaesthesia (as an extension of labour epidural). In addition to the reported case, our patient had a coexisting terminal filum lipoma on top of PMC which limited our type of central neuraxial technique and unfortunately suffered a paraparesis postoperatively which was eventually attributed to hypokalemic periodic paralysis.

Preoperatively, we were limited by time and the lack of definitive investigations in a pregnant patient presenting at full term. Understandably, she had declined repeat muscle biopsies and MRI scans. Nevertheless, we acted based on strong clinical suspicion and caution and consulted the valuable opinions of other specialist teams. Most types of myotonias are usually poorly classified or controversial at presentation. In addition, most terminal filum lipomas may often go undiagnosed. [6]

PMC predisposes a patient to myotonias exacerbated by exercise and cold. Perioperatively, we took meticulous care to ensure normothermia and avoid shivering. This was done with the help of warmed fluids, actively warming theatre environment and minimizing skin exposure at all times.

Lehmann et al. reported that there is no increased susceptibility to malignant hyperthermia in patients with PMC. [7] The rationale is that PMC mainly affects the sodium channels, and for that reason is distinct from the risks of malignant hyperthermia (MH) which is due to calcium channel abnormality. Yet, without prior clear classification of her condition, we erred on the side of caution and took special care to avoid any risks triggering MH, anesthesia induced rhabdomyolysis or suxamethonium induced hyperkalaemia.

Our preferred technique was regional anesthesia over general anesthesia. Regional anesthesia will help us avoid (1) the use of volatiles especially halothane, which commonly produced post op shivering, (2) the use of suxamethonium due to its hyperkalemic effect that can induce myotonias and malignant hyperthermia and (3) the use of neostigmine, which can potentiate myotonias.

Lipoma of the terminal filum is often an incidental finding of no clinical concern. However, in some patients who are symptomatic, it may be associated with tethered cord syndrome in which the conus is often low-lying and more posteriorly displaced within the spinal canal. [3] This is especially important given the recent evidence that anaesthesiologists tend to be at least one level higher than they think when performing a central neuraxial block. [8] Moreover, the syndrome may not be evident until a precipitating event such as child birth, following which regional anaesthesia may be implicated.

The general principles are that regional anesthesia is not contraindicated, although meticulous care regarding the following is required – (1) an epidural-only technique over a spinal is preferred in order to spare the dura in case there was any tethering of the spine; (2) to perform the block below the level of the conus or as low as possible; (3) Informed consent in these patients should include a discussion on the increased risk of spinal cord trauma; (4) Immediate removal of needle or catheter is mandated if there is pain on insertion; (5) a thorough pre anaesthesia neurological examination is important to mark the patient’s baseline signs.

Periodic paralysis (PP) is a rare muscle disease characterized by recurrent muscle weakness, occurring at a rate of 0.001 % [9] which may overlap with PMC as it maps to the same gene locus on chromosome 17. Generally, based on serum potassium levels, PP can be categorized into hypokalemia, normokalemia, and hyperkalemia subtypes. Ictal paresis is caused by depolarization of the muscle sarcolemma, which in turn causes sodium channel inactivation and reduced fibre excitability [9]. We ensured strict monitoring of potassium and minimized exogenous intake perioperatively. In this case, we allowed endogenous control of potassium intraoperatively, however found her to have a borderline low normal potassium post operatively which attributed the weakness. The usual treatment options include avoidance of triggers, supplementary potassium therapy or avoidance, followed by the use of carbonic anhydrase inhibitors [10].

This is a rare case of an obstetric patient with multiple anaesthetic challenges – a coexisting history of PMC and a terminal filum lipoma complicated by hypokalaemic periodic paralysis after an emergency CS under epidural. This case highlights the following learning points – (1) Proactive collaborative multi-disciplinary team effort is paramount and the assessment and counselling not only needs to be comprehensive but also within limits of an emergency setting and an obstetric patient. (2) Sometimes myotonias are not clearly classified on presentation especially during emergency situations. Regional anaesthesia for emergency CS will avoid the need for neostigmine, volatiles and suxamethonium which can exacerbate myotonias and potentially malignant hyperthermia. (3) A low conventional epidural block may help spare the dura due to potential tethering of the cord in terminal filum lipoma. (4) Ensuring normothermia helps to avoid cold related exacerbations of myotonia. (5) Even despite our best efforts to ensure normal physiological conditions perioperatively, the patient still presented with periodic paralysis with a borderline low normal potassium. It was largely expectant management with potassium supplementation, but during such times it is reasonable for patients to be worried, and it is important to provide continued support and reassurance whilst the patient is recovering.

## Data Availability

Not applicable.

## Abbreviations

PMC: Paramyotonia congenita
CS: Caesarean section
MRI: Magnetic resonance imaging
ECG: Electrocardiogram
PCA: Patient controlled analgesia
CSE: Combined spinal epidural
L2: Lumbar level 2
PP: Periodic paralysis
